# COVID-19: GENERAL GUIDELINES FOR CARDIOVASCULAR SURGEONS (standard guidelines - subject to change)

**DOI:** 10.21470/1678-9741-1-2020-0604

**Published:** 2020

**Authors:** Leila Barros, Luiz Antônio Rivetti, Beatriz Helena Furlanetto, Eduardo Miranda Teixeira, Alexandre Welikow

**Affiliations:** 1 Department of Cardiovascular Surgery, Santa Casa de Misericórdia de São Paulo, São Paulo, Brazil.; 2 Editor Fellow of the Brazilian Journal of Cardiovascular Surgery (BJCVS), São Paulo, Brazil.; 3 Department of Cardiovascular Surgery, Faculdade de Ciências Médicas, Santa Casa de São Paulo, São Paulo, Brazil.; 4 Director of the Instituto de Pesquisa, Inovação Tecnológica e Educação da Santa Casa de São Paulo (IPTEC), São Paulo, Brazil.; 5 Associate Editor of the Brazilian Journal of Cardiovascular Surgery (BJCVS), , São Paulo, Brazil.; 6 Hospital Infantil Sabará and ECMO Team Sabará, São Paulo, Brazil.; 7 Hospital Beneficência Portuguesa de São Paulo, São Paulo, Brazil.; 8 Instituto Furlanetto, São Paulo, Brazil.; 9 Santa Casa de Misericórdia de São Paulo, São Paulo, Brazil; 10 Enrolled at the Master’s Program of Public Health and Health Economy, University of Gothenburg, Sweden.; 11 Enrolled at the Master’s Program of Business Creation and Entrepreneurship in Biomedicine, Sahlgrenska School of Innovation and Entrepreneurship and Chalmers School of Entrepreneurship, Sweden.

We are experiencing a pandemic of the new Sars-Cov-2 coronavirus and its clinical presentation as a disease, the Covid-19. Since the first cases, recorded in December last year in the fish market in Wuhan, China^[1]^, Sars-Cov-2 has infected thousands of people around the world^[2]^, and with this, an avalanche of information and emotions has changed the world economy and the routine of many people. Important considerations should be given to our readers regarding cardiac surgery, surgical teams, our patients, and their possible contacts.

The identification of Covid-19 cases as a notifiable disease is guided by criteria that are constantly updated by the Brazilian Ministry of Health^[3]^. At the time of this editorial, those cases in which the patient presents with fever and respiratory symptoms of the common cold, cough, difficulty breathing, and nasal wing beat, among others, and which are associated with a recent history of travel to a region with local transmission, or of close contact with a suspected case, or of contact with a laboratory-confirmed case, in the 14 days prior to the appearance of signs or symptoms, are considered suspect cases. It is estimated that the average incubation period of Covid-19 is 5.2 days, which can reach 14 days. The average period of transmission of this disease is 5 days after the onset of symptoms, however, it is believed that transmission can occur even in the absence of symptoms. On the other hand, confirmed cases, regardless of clinical presentation, are those with confirmatory laboratory tests of Covid-19^[4]^.

The classification of Covid-19 as a pandemic implies the advancement of the social and health services response to the viral threat. The phase of early detection and containment of focal cases, previously related to travelers to areas at risk, becomes associated, with the beginning of community transmission in Brazil, with strategies to prevent deaths and the development of more serious cases in risk groups: the so-called mitigation phase. In this phase, the identification of the source case becomes more unlikely, and the tracking of contacts becomes more difficult.

This expansion of the strategy to combat Covid-19 is important in preparing for the exponential number of thousands of cases expected in the coming weeks in Brazil. Historically, some lessons have been taught by respiratory H1N1 influenza pandemics, such as the Spanish flu, of 1918, and the swine flu, of 2009, which are valid for Covid-19. Perhaps one of the most important lessons, the early social distancing has the greatest potential to delay the maximum expected case advance.

In addition to governmental measures, such as prohibition of agglomerations - that Brazil now adopts -, other measures directly affect the doctor-patient relationship and surgical planning. Indefinite cancellation of elective surgeries is already a reality in European countries, anticipating the growing demand for beds and the possible need for ventilatory support for Covid-19 patients. Likewise, the Brazilian Ministry of Health considers this strategy of elective surgery postponement in different centers. On the other hand, telemedicine and electronic solutions in health care have gained prominence; since the federal initiative of the Coronavirus - SUS app^[5,6]^, there has been an expansion of private medical consultation services by teleconference^[7]^.

For the surgeon, the choice of the surgical moment becomes an even more sensitive decision in times of Covid-19 pandemic. The lethality of this illness varies in age groups and is concentrated in individuals over 50 years old, with fatality rates ranging from 1.3% to 14.8% in individuals over 80 years old^[8]^. In hospitalized patients, the fatality rate is even higher, between 11% to 15%^[9]^. Of those infected, 10% are expected to be severe cases and 5% should require admission to the Intensive Care Unit^[9]^. In a study published in The Lancet journal comparing surviving cases with deaths, elevated ultra-sensitive troponin 1 was found in more than half of the deaths, contributing to the findings of acute cardiac injury in up to 12%^[10]^ of the cases.

Lung injury is the most common complication in these patients. In the same abovementioned study from The Lancet, in the non-survival group, 98% had respiratory failure, 50% secondary infections, and 100% sepsis^[11]^. In all Covid-19 patients, the most striking condition is severe acute respiratory syndrome (SARS), which can affect 17%-29% of them^[9,12,13]^. Additionally, 75% of the patients present with atypical bilateral pneumonia and 14% with bilateral ground-glass opacity, which is better seen on computed tomography images than on chest X-rays^[9]^.

In selected SARS patients, early orotracheal intubation is recommended. Early intubation is important both in patients who do not respond well to noninvasive ventilation and as a strategy for infectious contingency, with the sealing of the invasive ventilation system.

In a report by the World Health Organization, as an alternative to the refractory response to conventional treatment in patients with persistent hypoxemia on mechanical ventilation, there is also the recommendation to use Extracorporeal Membrane Oxygenation (ECMO). The reduced mortality achieved with ECMO in SARS patients is already documented in different studies. In an article published in the Journal of the American Medical Association (JAMA), there is also the recommendation of early transfer of critical Covid-19 patients to specialized ECMO centers, in order to decrease organic deterioration before the installation of ECMO.

Presence of comorbidities, advanced age, number of days on invasive mechanical ventilation, and clinical evolution of the patient serve as a reference for the indication of ECMO, especially the venous-venous type. The risk of complications and unfavorable outcomes for these patients should always be compared to the indication of ECMO for each case. It is important to note that SarsCov-2, when causing acute myocarditis with acute heart failure, may, however, lead to the indication of venoarterial ECMO.

The ECMO group at the University Hospital Zurich, Switzerland, led by Dr. Maximilian Halbe published its recommendations for indication and contraindication of ECMO in Covid-19 patients. These are indications for venous-venous ECMO: patients with SARS, Murray Score for acute lung injury ≥ 3, and Horowitz index (PaO2/FiO2) < 50-70 mmHg, in positive end-expiratory pressure > 15 cmH2O. For venoarterial ECMO, the indications are: cardiogenic shock with progressive increase in lactate despite the association with inotropes and vasopressors and cardiac index < 2. These are contraindications for ECMO in Covid-19 patients: advanced age (> 70-75 years), depending on related clinical criteria; pH < 6.8 or Murray score < 3; poor-prognosis neoplasm; multiple organ failure with no cardiac or pulmonary cause; and relative contraindication for chronic diseases.

ECMO is an important therapeutic resource for Covid-19 patients, despite the lack of consensus among specialists for this specific indication.

The Extracorporeal Life Support Organization (ELSO) is in the process of acquiring data to formulate its official recommendations. ECMO installations must be carried out by specialists in qualified centers with large experience in this procedure.

In times of exponential Covid-19 infections and growing demand for health services, the surgeon has an important role in reinforcing the good health practices defined by the Brazilian Ministry of Health to the general population. These are: wash your hands with soap and water; avoid touching your face with your hands; do not share personal items; use alcohol gel on your hands; and vaccinate against seasonal influenza, especially patients with major comorbidities.
